# Increasing Accessibility of Bayesian Network-Based Defined Approaches for Skin Sensitisation Potency Assessment

**DOI:** 10.3390/toxics12090666

**Published:** 2024-09-12

**Authors:** Tomaz Mohoric, Anke Wilm, Stefan Onken, Andrii Milovich, Artem Logavoch, Pascal Ankli, Ghada Tagorti, Johannes Kirchmair, Andreas Schepky, Jochen Kühnl, Abdulkarim Najjar, Barry Hardy, Johanna Ebmeyer

**Affiliations:** 1 Edelweiss Connect GmbH, Hochbergerstrasse 60C, 4057 Basel, Switzerland; tomaz@edelweissconnect.com (T.M.); andrii@edelweissconnect.com (A.M.); artem@edelweissconnect.com (A.L.); pascal@edelweissconnect.com (P.A.); ghada@edelweissconnect.com (G.T.); 2 Beiersdorf AG, Beiersdorfstraße 1-9, 22529 Hamburg, Germany; anke.wilm@beiersdorf.com (A.W.); stefan.onken@beiersdorf.com (S.O.); andreas.schepky@beiersdorf.com (A.S.); jochen.kuehnl@beiersdorf.com (J.K.); 3 Department of Pharmaceutical Sciences, Division of Pharmaceutical Chemistry, Faculty of Life Sciences, University of Vienna, Josef-Holaubek-Platz 2, 1090 Vienna, Austria; johannes.kirchmair@univie.ac.at

**Keywords:** Bayesian network, defined approaches, next-generation risk assessment, NAMs, skin sensitisation

## Abstract

Skin sensitisation is a critical adverse effect assessed to ensure the safety of compounds and materials exposed to the skin. Alongside the development of new approach methodologies (NAMs), defined approaches (DAs) have been established to promote skin sensitisation potency assessment by adopting and integrating standardised in vitro, in chemico, and in silico methods with specified data analysis procedures to achieve reliable and reproducible predictions. The incorporation of additional NAMs could help increase accessibility and flexibility. Using superior algorithms may help improve the accuracy of hazard and potency assessment and build confidence in the results. Here, we introduce two new DA models, with the aim to build DAs on freely available software and the newly developed kDPRA for covalent binding of a chemical to skin peptides and proteins. The new DA models are built on an existing Bayesian network (BN) modelling approach and expand on it. The new DA models include kDPRA data as one of the in vitro parameters and utilise in silico inputs from open-source QSAR models. Both approaches perform at least on par with the existing BN DA and show 63% and 68% accuracy when predicting four LLNA potency classes, respectively. We demonstrate the value of the Bayesian network’s confidence indications for predictions, as they provide a measure for differentiating between highly accurate and reliable predictions (accuracies up to 87%) in contrast to low-reliability predictions associated with inaccurate predictions.

## 1. Introduction

Skin sensitisation is characterised by four main molecular and physiological Key Events (KEs) [1]. These KEs include (i) the covalent binding of a chemical to skin peptides and proteins (KE1); (ii) the activation of keratinocytes, which constitute the main cell type in skin (KE2); (iii) the activation of dendritic cells (KE3); and (iv) the activation of T-cells (KE4) [2]. Repeated exposure to a compound can result in contact dermatitis. The Next-Generation Risk Assessment (NGRA) of skin sensitisation potential is based on integrating in silico predictions and in vitro test results in defined approaches (DAs). DAs integrate data from various sources, i.e., in vitro, in chemico, and in silico, to support skin sensitisation assessment using a predefined set of resources and data interpretation [3]. Thus, DAs have the potential to align with the principles of the OECD Mutual Acceptance of Data (MAD) agreement. This agreement requires that data generated for regulatory purposes in one member country be recognised and accepted by other OECD member countries [4]. DAs can further be used to support skin sensitisation assessment within Integrated Approaches to Testing and Assessment (IATAs). IATAs use the weight of evidence (WoE) and expert evaluation to support decision-making for regulatory bodies [5].

In 2017, two guidance documents on the reporting of DAs (OECD GD 255 and OECD GD 256) were released [3,6]. In 2018, the U.S. EPA issued a draft science policy document reporting two DAs: two out of three (2o3) and the Key Event 3/1 Sequential Testing Strategy (KE 3/1 STS) [7]. In 2021, the OECD published guideline 497 on defined approaches to skin sensitisation, which incorporated three DAs: the “2o3” DA relies on two concordant tests from three in vitro assays addressing the three first KEs, namely, the Direct Peptide Reactivity Assay (DPRA, KE1), KeratinoSens^TM^ (KE2), and the human Cell Line Activation Test (h-CLAT, KE3) [1]. Furthermore, guideline 497 features two versions of ITS (ITSv1 and ITSv2), which integrate the results of DPRA and h-CLAT with in silico predictions. The primary distinction between these two versions is the use of different in silico tools. ITSv1 uses the Derek Nexus software, whereas ITSv2 incorporates the OECD QSAR Toolbox. In addition, both ITS versions provide a scoring system to predict potency classification based on the United Nations Globally Harmonised System of Classification and Labelling of Chemicals (GHS) [8]. Edelweiss Connect recently developed the SaferSkin approach, a solution package for skin sensitisation risk assessment [9]. The SaferSkin suite offers and applies OECD DAs, IATAs, and new approach methodologies (NAMs) to perform animal-free risk assessment of substances. The solution combines a variety of OECD-required standard in vitro assays with a set of in silico prediction tools based on DAs, machine learning, and Quantitative Structure–Activity Relationship (QSAR) approaches [1,10,11].

In this article, we present a new DA based on the previously reported Bayesian network (BN) ITS-3 model of Jaworska et al. by reflecting the advances in both in vitro experiments and in silico modelling [11,12]. The aim of the study is to increase the transparency and applicability of the available models by integrating a new in vitro assay and replacing commercial in silico tools with freely available ones. A BN is a powerful tool for representing complex relationships between variables. It employs a visual map with nodes depicting factors of interest and arrows indicating their causal or influential connections. Conditional probability tables associated with each node quantify the likelihood of one factor occurring based on the states of others. This allows us to analyse the interplay of variables and make probabilistic inferences within a particular domain. BNs are particularly valuable due to their capacity to elucidate connections among multiple factors, which may not be readily apparent, even to domain experts. In addition, BNs provide a probabilistic assessment of skin sensitisation by integrating diverse data sources to form evidence-based hypotheses and are designed to handle incomplete data easily. Notably, BNs also enable the evaluation of prediction uncertainties based on the quality and completeness of the input data. The primary goals of the present work were to incorporate additional NAMs and to improve the BN model’s accessibility. Hence, BN models were developed using public and in-house-generated data and available open-source software. Data used for modelling and evaluation were collected from the literature and carefully curated; new kinetic DPRA (kDPRA) data were also generated to fill data gaps (Tables S1 and S2 in Supplementary Materials). Edelweiss Connect developed a BN model for skin sensitisation assessment based on the previously reported BN model by Jaworska et al. [9,11,12]. The BN model developed by Edelweiss Connect is referred to as “SaferSkin-BN” in this publication. SaferSkin-BN was shown to have similar predictive characteristics to the original model developed by Jaworska [13]. This article describes the changes implemented in the SaferSkin-BN model, which include the incorporation of kDPRA parameters and using inputs from publicly accessible Skin Doctor CP and the open-source software OPERA [14,15]. The original model from Jaworska et al. and SaferSkin-BN use commercial software (TIMES-SS and ACD/Labs) [16,17]; we investigated the impact of the replacement of the commercial software by Skin Doctor CP and OPERA on model performance. Since potency estimation is one of the main challenges in skin sensitisation assessment, we introduced the kDPRA as a new in vitro assay input parameter. To date, the kDPRA is the only OECD-validated in vitro assay allowing for the classification of skin sensitisers according to the Globally Harmonised System (GHS) classes 1A and 1B/non-classified [18].

In silico toxicology protocols demand the combined use of rule-based and statistics-based models [19]. In order to appreciate this concept, we developed a second model that was able to incorporate additional in silico prediction models, i.e., Derek Nexus. We aimed to either improve the predictive performance and include a specific evaluation of uncertainty and potency or to maintain the original model performance but increase accessibility, which can be used to support risk assessment goals. Thus, we adjusted the SaferSkin-BN model by implementing the following modifications:

1. The use of kDPRA data with the rate constant Kmax and GHS class prediction in addition to DPRA data as input parameters.
2. The replacement of the quinary in silico predictions of skin sensitisation by TIMES-SS with the binary and ternary predictions of Skin Doctor Conformal Predictor (Skin Doctor CP).
3. The replacement of the in silico predictions of physicochemical properties by ACD/Lab with those by OPERA.
4. The extension of the BN model with a rule-based system, i.e., Derek Nexus [20].

The developed BN models were trained by assessing different hyperparametrisation options and tested to determine their predictive performance. In addition, the influencing inputs on the final outcomes were evaluated as follows:

- Using a BN with ternary Skin Doctor CP and kDPRA to assess the predictive performance of the Skin Doctor CP prediction vs. kDPRA;
- Implementing the Skin Doctor CP *p*-value;
- Using ternary Skin Doctor CP, kDPRA, and Derek Nexus results;
- Assessing the impact of using DPRA and kDPRA.

The current work presents different models based on the proposed modifications. Thus, several models were tested in this approach, and the best-performing ones were incorporated into the SaferSkin software (Edelweiss Connect GmbH, Basel, Switzerland). The performance of the newly developed models was assessed based on a predefined test set (Cosmetics Europe database) that was used previously to evaluate the performance of DA models [21,22]. SaferSkin-BN served as a baseline model for comparison.

## 2. Materials and Methods

### 2.1. In Vivo Inputs: LLNA

Regulatory authorities have accepted the Local Lymph Node Assay (LLNA, OECD 429) as a high standard for evaluating skin sensitisation hazards and risks [23,24,25,26]. The LLNA assay is based on the induction of lymphocyte proliferation in the lymph nodes by draining the site of the test substance’s application. This proliferation is proportional to the dose and potency of the applied allergen and provides a simple means of obtaining a quantitative measurement of sensitisation (OECD 429). This includes the activation and proliferation of antigen-specific T-cells [27]. Skin sensitisation potential is attributed to a chemical if the proliferation rate exceeds a factor of 3 compared with the vehicle-treated control group. The concentration estimated to stimulate a three-fold increase in lymphocyte proliferation (termed “EC3”) refers to the threshold concentration, which provides information on the chemical’s skin-sensitising potency. Originally, the concentration in LLNA was reported on a “per-weight basis”, but for modelling purposes, it was transformed into molar units using the formula pEC3 = log(Mw/250/EC3(%)) [12]. The following pEC3 thresholds were applied to derive four potency classes in molar units: non-sensitiser (<−1.9), weak sensitiser (−1.9, −1.1), moderate sensitiser (−1.1, −0.35), and strong sensitiser (>−0.35) [12]. Thus, the formed variable, the pEC3 class, was the target variable in the BN model. The LLNA data used to develop the model described here were collected from Jaworska et al. [12].

### 2.2. In Vitro Inputs

#### 2.2.1. DPRA

The DPRA addresses KE1 of the AOP for skin sensitisation, i.e., protein binding, and it determines the reactivity of a test compound towards synthetic cysteine- and lysine-rich peptides [18]. Peptide reactivity is reported as a percentage of peptide depletion. For the DA described by Jaworska et al., the percentage of free peptide remaining in the sample is used for all calculations. For developing and applying the models described in this publication, the percentage depletion was used as the input parameter and calculated with respect to the percentage of the peptide remaining. The DPRA data used to develop the model described here were collected from Jaworska et al. [12].

#### 2.2.2. kDPRA

The kDPRA is currently the only OECD-accepted in vitro/in chemico assay that was reported to enable skin sensitisation potency classification [18]. This assay is a modification of the DPRA. It allows the derivation of a rate constant (Kmax (in 1/sM)) of the depletion of the cysteine-containing peptide upon reaction with the test compound. Depending on the rate constant, a test compound can then be classified as GHS 1A or GHS 1B/not classified. The data were partly derived from studies conducted by Natsch et al. and Natsch and Gerberick [28,29]. For four compounds out of these databases, one study reported an unambiguous result while the other one reported no clear study result. In those cases, the unambiguous result was included in our dataset. No conflicting results were observed between the two data sources. Additionally, new kDPRA data were generated. The kDPRA was performed as described in OECD guideline 442C and in the full method description in the database service on alternative methods (DB-ALM) in protocol 217 [18,30]. The cysteine peptide was provided by RS Synthesis (Louisville, KY, USA). The chemicals were purchased from Sigma-Aldrich (St. Louis, MO, USA) or Alfa Chemistry (Holbrook, NY, USA). The assay was run in 96-well plates. The chemicals were dissolved at 20 mM in acetonitrile (ACN), diluted in acetonitrile, and added to the cysteine peptide solution (0.66 mM in phosphate buffer) to final concentrations of 5, 2.5, 1.25, 0.625, and 0.3125 mM. Monobromobimane (3 mM in ACN) was added after 10, 30, 90, 150, 210, and 1440 min, respectively, and the fluorescence intensity was measured at Ex/Em 390/480 nm. Peptide depletion and Kmax were evaluated using an Excel spreadsheet provided as Supplementary Material in DB-ALM 217 [30]. Not all compounds with missing kDPRA could be tested due to limited availability or solubility.

#### 2.2.3. KeratinoSens^TM^

The KeratinoSens^TM^ assay (OECD 442D) addresses KE2 of the AOP for skin sensitisation, i.e., the activation of keratinocytes. The assay evaluates the activation of the Keap1-Nrf2-ARE pathway, a key pathway triggered by sensitisers in vivo [31]. The average concentrations (in µM) leading to 1.5-fold and 3-fold induction (EC1.5 and EC3) are reported alongside the concentration leading to 50% cytotoxicity after 24 h (IC50). The data were collected from Jaworska et al. [12].

#### 2.2.4. h-CLAT

The h-CLAT assay addresses KE3 of the AOP for skin sensitisation (OECD guideline 442E), known as dendritic cell activation. The assay quantifies changes in the expression of cell-surface molecules (CD54 and CD86) [18]. Both molecules are essential in the induction of skin sensitisation; CD54 is involved in dendritic cell migration, and CD86 stimulates T-cell activation during antigen presentation to dendritic cells [32]. The average compound concentrations (in µM) inducing 150% of vehicle control expression of CD86 (EC150) and 200% of vehicle control expression of CD54 (EC200) are reported alongside the concentration leading to 75% cell viability after 24 h (CV75). The data were collected from Jaworska et al. [12].

### 2.3. In Silico Inputs

#### 2.3.1. OPERA

OPERA (v2.9) is a free and open-source suite of QSAR models providing predictions for physicochemical properties, environmental fates, and toxicity endpoints [15,33]. OPERA was used to calculate the octanol–water distribution coefficient of compounds at pH 7 (logD@pH7), the water solubility at pH 7 (Ws@pH7), plasma–protein binding, and the octanol–water partition coefficient (log Kow). The fraction ionised (fion) was calculated using the formulation = |1 − 10^logD/10^logKow|, where “||” denotes the absolute value [12].

#### 2.3.2. Skin Doctor CP

Skin Doctor CP is a conformal prediction-based machine learning model for the classification of small organic compounds into two (non-sensitisers and sensitisers) or three (non-sensitisers, weak to moderate sensitisers, and strong to extreme sensitisers) potency classes [34]. The models were trained on publicly available data only. They were transparently published and are accessible via a web service [35]. As a conformal prediction model, Skin Doctor CP will only return a prediction if predefined reliability thresholds are met, and these can be adjusted for the specific use case.

#### 2.3.3. Derek Nexus

Derek Nexus (v6.3) (Lhasa Limited, Leeds, UK) is a rule-based commercial modelling software that can predict the likely toxicity of a given chemical structure based on the absence or presence of certain chemical substructures [20]. It has also been recently included in the DA for skin sensitisation published by the OECD [1]. For skin sensitisation, it is one of only a few models that can predict not just hazards but also EC3 values. These values can then be transferred into five distinct potency classes: non-sensitisers and weak, moderate, strong, and extreme sensitisers. For the modelling, we merged the strong and extreme sensitiser classes into one class.

### 2.4. Bayesian Network-Based Defined Approach

#### 2.4.1. Dataset

The starting point for model development was the dataset collected by Jaworska et al., which included LLNA, DPRA, KeratinoSens^TM^, and h-CLAT data, as well as calculated physicochemical properties for 207 compounds [12]. We extended the dataset with kDPRA data from two sources, as well as with newly generated kDPRA data plus sensitiser potency predictions from Skin Doctor CP and Derek Nexus (which replaced TIMES-SS potency predictions in the original dataset) [28,29]. Additionally, the physicochemical parameters originally calculated using commercial software were newly calculated using the open-source OPERA suite. The original split into training (147 compounds) and test sets (60 compounds) was preserved from Jaworska et al. to ensure a fair model comparison [12]. The dataset published by Jaworska et al. included two compounds (Farnesol and Benzyl cinnamate) that are present in training and test set at the same time but each time with different SMILES and a different set of parameters present. Due to comparability we decided to not change the data. The full training and test set used for model building and evaluation within this study can be found in Supplementary Materials Tables S1 and S2, respectively.

#### 2.4.2. Discretisation

BNs require discretised continuous variables. Hence, continuous input variables were discretised into four classes using a supervised clustering algorithm (class–attribute interdependence maximisation, CAIM) implemented in the R package discretisation [36,37]. Discrete variables (potency classes from Skin Doctor CP, Derek Nexus, and kDPRA classification) were not modified.

#### 2.4.3. Building Latent Nodes

In addition to the observed variables (i.e., in vitro and in silico inputs), we used latent variables in the network structure. These variables were not directly observable but simplified the network structure (reduced connections between the nodes) and often improved the model accuracy. Latent variables were designed in a way that they connected similar input variables. These latent nodes were built in the training set by grouping the connected input variables into a predefined number of classes. This grouping was essentially an unsupervised clustering performed through latent class analysis (LCA) using an R package named poLCA: Polytomous Variable Latent Class Analysis [38]. This clustering is essentially a search for a global maximum of the log-likelihood function, so, understandably, its outcome may depend on the initial conditions and number of repetitions. For the test set, the latent nodes were empty, and their values were inferred during the BN prediction.

#### 2.4.4. Bayesian Network

A Bayesian network is a powerful tool to model probabilistic knowledge about certain phenomenon. Nodes in the network represent discrete variables that are connected with arrows. Each arrow represents a conditional probability table (network parameters) between the two connected variables. Bayesian networks allow one to calculate the probability distribution of unobserved discrete variables given the evidence (i.e., values of observed variables).

**Figure 1:**
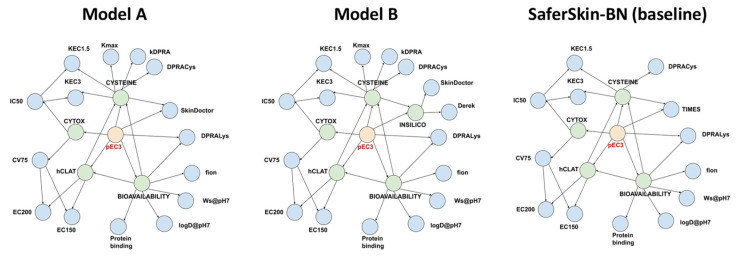
Architecture of the three Bayesian networks (BNs): model A (**left**), model B (**middle**), and the baseline SaferSkin-BN model (**right**). Blue nodes denote input parameters; green nodes represent latent variables; red nodes designate the target variable (pEC3 class).

Building a BN model involves two steps: (i) defining the network structure (i.e., connectivity among the nodes) and (ii) learning the parameters of the network (i.e., conditional probability tables). There were many possible connections among the nodes, and it was not our intention to ascertain the one giving the highest possible performance. Instead, we relied on the BN structure proposed by Jaworska et al., which was developed manually from mechanistic knowledge of skin sensitisation [12]. We tested two different BN structures (Figure 1) that both largely resembled the BN architecture from SaferSkin-BN (or Jaworska et al. [12]) with the following modifications: (i) parameters from the kDPRA were connected in the same way as the DPRACys (i.e., the percentage of cysteine peptide depletion in the DPRA) variable; (ii) the in silico TIMES-SS input was replaced by the Skin Doctor CP (left network) or by the newly formed latent variable INSILICO, which connected the inputs from Skin Doctor CP and Derek Nexus (right network in Figure 1). Once the BN architecture was defined, its parameters (conditional probability tables) were learned from the training set. For the BN modelling, we used the Python library pgmpy and the following settings: estimator = BayesianEstimator, prior_type = K2, equivalent_sample_size = 1 or 5, complete_samples_only = False [39].

The BN model predicted discrete probability distributions of all the unknown variables. For the test set, the unknowns were the target variable, latent nodes, and possibly some input nodes. However, due to the non-uniform class distribution of target variables in the training set, the resulting probability was biassed towards the more populated classes. To correct this bias, we followed the approach presented by Jaworska et al. and calculated Bayes factors [12]. Then, the predicted class was the one with the highest Bayes factor. The size of the Bayes factor was also directly related to the confidence in a prediction [40]: with Bayes factor < 3.2 indicating weak confidence, 3.2 ≤ Bayes factor < 10 indicating substantial confidence, 10 ≤ Bayes factor < 32 indicating strong confidence, and Bayes factor > 32 indicating very strong confidence. With latent class analysis, we introduced several hyperparameters that required optimisation: (i) each latent node had a defined number of classes; we tested three to five classes for each node; (ii) the number of repetitions of latent class analysis could also influence the BN model performance; we tested three values, i.e., 10, 20, and 50. We performed an extensive grid search in the space of hyperparameters and selected the set of hyperparameters that resulted in the highest balanced accuracy on the test set. Balanced accuracy was calculated using the method *sklearn.metrics.balanced_accuracy_score* from the *scikit-learn* Python library version 1.5.1. The method calculates balanced accuracy as an average recall over the four potency classes [41].

During hyperparameter optimisation, we selected a set of hyperparameters for each network structure, leading to the highest balanced accuracy on the test set. However, this outcome could require further investigation to avoid overfitting the test set by the hyperparameters. Hence, we estimated the accuracy of the two best models using 10-fold cross-validation. The full dataset (training set + test set) was randomly split into 10 folds. Nine folds were used to train the discretisation scheme, run latent class analysis (just with the set of hyperparameters determined during hyperparameter optimisation in Section 3.1), and train the BN parameters. The trained model was used to generate predictions for the 10th fold.

#### 2.4.5. Evaluation Dataset

The performance of model A and model B in comparison with the baseline SaferSkin-BN model was evaluated using the Cosmetics Europe Database, a dataset compiled by the Cosmetics Europe consortium [21,22]. It was previously used by Kleinstreuer et al. to evaluate the performance of different DA models [22]. The dataset covers, e.g., in vitro data (DPRA, KeratinoSens^TM^, h-CLAT) and LLNA data for 128 substances. The dataset was prepared and curated to include additional required in silico and in vitro data for the current modelling approach; physicochemical parameters were generated using OPERA. Derek Nexus and Skin Doctor CP were used to generate predictions for the INSILICO nodes in model A and model B. kDPRA data were collected from Natsch and Gerberick [29]. TIMES-SS data for comparison with the SaferSkin-BN were collected from Hofmann et al. [42]. Of the 128 substances in the original dataset, 6 were of natural origin with unknown exact compositions. We had to omit those 6 compounds as converting mass concentrations into molar concentrations or deriving a defined chemical structure for in silico predictions was impossible. The full evaluation set is summarised in Supplementary Materials Table S3.

## 3. Results and Discussion

The current work presents the development of novel BN models prioritising transparency and reproducibility to address regulatory concerns and support future regulatory acceptance. The starting point for model development was SaferSkin-BN, based on the ITS-3 BN approach developed by Jaworska et al. [12]. The Jaworska ITS-3 model uses two commercial software packages, TIMES-SS (for predicting skin sensitisation potency) and ACD/Labs (for predicting physical–chemical properties), as well as in vitro input parameters (DPRA, KeratinoSens^TM^, and h-CLAT). SaferSkin-BN was built using OPERA, TIMES-SS, DPRA, KeratinoSens^TM^, and h-CLAT.

Within the present study, we successfully added a new in chemico assay, kDPRA, which was also recently included in the updated OECD guideline 422C on assays addressing KE1, i.e., covalent binding to proteins. In addition, the successful replacement of commercial in silico tools by freely available ones could be shown.

We developed two slightly different models, models A and B, incorporating these two steps. The first step involved adding two input nodes to the BN representing data from the kDPRA, i.e., Kmax and kDPRA classification according to GHS classes. Both nodes were considered equivalent to the DPRACys node representing the cysteine depletion value determined in the DPRA and were therefore connected to the CYSTEINE latent node just like DPRACys. This pattern was the same in both model A and model B.

These changes resulted in model A, which is based solely on freely accessible in silico tools.

Another variant was developed to complement the statistics-based Skin Doctor CP model with a rule-based in silico model and sensitisation potency prediction. To this end, we integrated Derek Nexus as an additional input. While we strived to use open-access tools, the inclusion of Derek Nexus was favourable for considering the recommendations for in silico toxicology protocols due to its recent adoption in the OECD guideline 497 on defined approaches on skin sensitisation and to improve the performance with respect to that of model A [1,19]. To reduce connectivity in the BN and optimise its performance, we created a new latent node, INSILICO, that replaced the TIMES node in SaferSkin-BN. The new INSILICO node was connected to the two in silico inputs from Skin Doctor CP and Derek Nexus. The resulting architecture represented model B.

### 3.1. Hyperparameter Optimisation

After determining the final network structures, we ran an extensive hyperparameter search to maximise the balanced accuracy on the test set. Hyperparameters refer to the latent class analysis during data preparation. In the latent class analysis, we assumed the number of classes (N_CYTOX_, N_CYSTEINE_, N_HCLAT_, N_BIOAV_, N_INSILICO_) for each latent node (CYTOX, CYSTEINE, HCLAT, BIOAV, INSILICO). Furthermore, we assumed the number of times the global optimum is estimated using different initial conditions, NREP. The space of these six hyperparameters was extensively searched, and Table 1 summarises the optimal set of parameters found for models A and B. Further results reported in this section are based on the analysis of those optimised versions of models A and B in comparison with SaferSkin-BN.

### 3.2. Incorporation of kDPRA into In Vitro Parameters

The OECD recently updated test guideline 422C on assays addressing KE1, i.e., covalent binding to proteins, with a new kDPRA. The kDPRA shows promising predictive capacity for risk assessment, distinguishing between GHS 1A-classified sensitisers and GHS 1B/non-sensitisers with good accuracy [18]. More kDPRA data might become available in the public domain in the future or might be generated during a specific compound’s risk assessment. Therefore, in the present work, we aimed to broaden the scope and source of the in vitro parameters that could be considered for DA.

The main parameter obtained in the kDPRA was Kmax, a rate constant for the binding of the compound to the peptide. The assay also defines a threshold in Kmax that best distinguishes between GHS 1A sensitisers and GHS 1B/non-sensitisers. Therefore, this assay provided us with two input parameters for the BN model: Kmax (a continuous variable that we discretised as described in the Methods section) and kDPRA (a binary variable representing the classification into GHS 1A or GHS 1B/non-sensitiser). Table 2 compares BN models with and without kDPRA input parameters. Balanced accuracies for the test set were calculated using four model variants (i.e., models A and B, each with and without kDPRA nodes). The individual predictions for all compounds from the test set by the baseline model, as well as model A and model B, can be found in Supplementary Materials Table S4.

Including the kDPRA data resulted in the comparable or even slightly improved predictive capacity of the BN model, thus enabling the use of the model with kDPRA data, DPRA data, or both, depending on their availability. In the field of skin sensitisation prediction, where relatively sparse data are available in the public domain, this flexibility will offer an advantage, especially if we expect the kDPRA to be conducted more frequently in the future due to its easier and faster application and its ability to predict a measure of potency.

### 3.3. Implementation of In Silico Skin Sensitisation Prediction Models

One critical parameter of the Jaworska ITS-3 model and SaferSkin-BN is the in silico prediction model of skin sensitisation potency, i.e., the TIMES-SS (TImes MEtabolism Simulator) V.2.27.13 commercial software [43]. TIMES-SS is a hybrid expert system that encodes structure–activity and structure–metabolism relationships and is therefore capable of predicting the sensitising potency of a parent compound, as well as that of its metabolites. In their analysis, Jaworska et al. demonstrated that this parameter had the highest predictive power of all the input parameters [11].

Within the present study, TIMES-SS inputs were replaced with predictions from the Skin Doctor CP model, which was transparently published in the literature, was trained on public LLNA data, and is available via a web service [35]. Table 3 compares the accuracies of BN models incorporating TIMES-SS or Skin Doctor CP inputs. In model A, we directly replaced the TIMES-SS input node with the Skin Doctor CP node. However, in model B, we added one more in silico parameter, i.e., the prediction of skin sensitisation potency from the Derek Nexus software. Hence, in model B, we actually created a new latent node, INSILICO, that connected both in silico inputs and replaced the TIMES-SS node.

The statistical analysis showed that replacing TIMES-SS with Skin Doctor CP maintained the model’s prediction accuracy. Addition of Derek Nexus predictions (model B) slightly increased the balanced accuracy. TIMES-SS predicted the skin sensitisation potential for the parent compound and predicted metabolites. The most conservative input was provided to the DA. Our newly generated models A and B were based on Skin Doctor CP and Derek Nexus. Both tools predicted the skin sensitisation potential based on the parent compound. Metabolites were not predicted. The impact of a potential sensitising metabolite was only indirectly considered, as these models were trained on LLNA in vivo data, which covered the effect of native, although not human, skin metabolism and, thus, captured the effects of both the parent compound and the metabolites formed. The impact of using in silico predictions for the metabolites on the overall performance of the model still needed proper evaluation. The superior predictive performance of model B over model A demonstrated a high impact of the in silico tool Derek Nexus on improving the model’s accuracy. This was aligned with the observation by Jaworska et al. that the in silico input (TIMES-SS in their case) was the input parameter with the largest impact on the final prediction [12].

### 3.4. Replacement of Physicochemical Parameter Prediction Models

In the Jaworska ITS-3 model, the physicochemical parameters (octanol–water partition coefficient, water solubility, fraction of compound ionised, protein binding) were calculated with the commercial QSAR models from ACD/Labs [12,17]. To increase the accessibility and usability of the DA, we calculated these parameters using the open-source OPERA suite. Table 4 summarises a head-to-head comparison of the newly developed models trained with physicochemical properties coming from ACD/Labs and OPERA while keeping the rest of the parameters unchanged (the network architecture was as depicted in Figure 1). Note that we determined the best set of hyperparameters for each of the two variants of model A (trained with ACD/Labs and trained with OPERA phys-chem). Incidentally, the same set of hyperparameters gave the highest test set accuracy for both model variants.

Replacing the physicochemical parameters from the commercial QSAR models with those from the open-source suite did not decrease the model’s accuracy.

### 3.5. Model Cross-Validation

After the optimal hyperparameters were determined, we ran a 10-fold cross-validation on the entire dataset (i.e., merged the training and test sets) to obtain a more realistic estimate of the model’s accuracy. Table 5 shows the cross-validated balanced accuracy (full dataset) of the two models, and these were clearly comparable to the balanced accuracies on the test set (Table 1). Furthermore, we calculated the minimum, median, and maximum balanced accuracy per fold to illustrate the variability in balanced accuracy on small datasets (a typical fold contained 20 compounds). We can see that the balanced accuracy was difficult to estimate with a relatively small set of compounds.

To illustrate the robustness of the choice of hyperparameters, we ran a 10-fold cross-validation for every set of hyperparameters and collected the minimum, average, and maximum balanced accuracy obtained. The results are presented in Table 6. We can conclude that the hyperparameters selected based on the balanced accuracy of the test set gave close to the best cross-validated balanced accuracy, too. Another observation was that we could expect about 10 to 15% variation in balanced accuracy between the best-performing and the worst-performing sets of hyperparameters. Expectedly, this variation was substantially lower than the variation in balanced accuracy among the folds.

### 3.6. Relation between the Prediction Confidence and Accuracy

An advantage of a BN is its ability to predict sensitiser potency alongside an indication of prediction confidence. More concordant evidence supports a particular prediction with a higher confidence level than non-concordant evidence [12]. To illustrate the influence of the confidence level on prediction accuracy, the accuracy of predictions with different confidence levels was calculated (Table 7). Importantly, accuracy improved substantially as confidence levels progressed from weak to very strong.

### 3.7. Prediction of the LLNA EC3 Value for Use in Risk Assessment

Although the proposed BN models provide discrete probability distributions, it is possible to derive a predicted continuous LLNA EC3 value from those distributions. This is of substantial value for any quantitative risk assessment. To the authors’ current knowledge, only a few approaches (i.e., the BN, the neural network, and the multiple linear regression approaches) predict EC3 values [44,45]. The discrete probability distribution over potency classes could be turned into a distribution over pEC3 values (by applying the pEC3 thresholds to the class limits). The most likely pEC3 value is the value at the 50th percentile of that distribution. However, it reports more restrictive values, e.g., at the 70th or the 90th percentile, which might be considered. The currently developed BN reports the probability distribution of pEC3, unlike the existing two DAs, which simply deliver a pEC3 value without a confidence interval.

**Figure 2:**
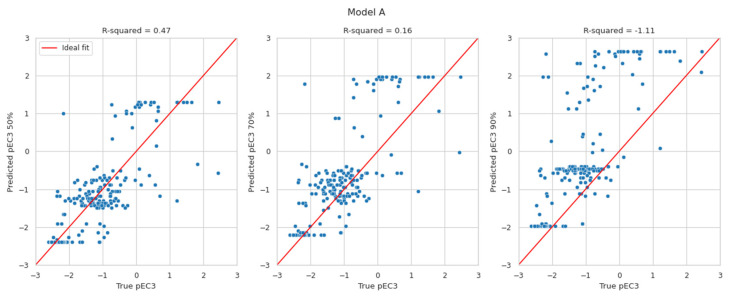

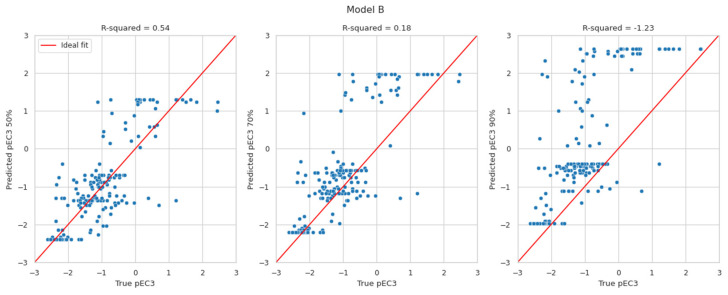
Predicted pEC3 taken at the 50th (**left**), 70th (**middle**), and 90th percentiles (**right**) versus the true pEC3 values for models A and B.

The results in Figure 2 display the predicted pEC3 values at the 50th (left), 70th (middle), and 90th percentiles (right) versus the true pEC3 values. The red lines indicate a perfect fit. The pEC3 values derived from the 50th percentile are scattered around the red line. Deriving pEC3 values from higher percentiles resulted in overestimating the potency (predicting lower EC3 values than the true LLNA results). Within expectations, the highest R^2^-values can be found for the 50th percentile. With values of 0.47 and 0.54, they are in line with the R^2^-values evaluated for Natsch et al., which are 0.53, 0.52, and 0.54 for EQ1, EQ4 and EQ5, respectively. Small R^2^-values for the 70th and 90th percentile, of course, result from the intention to avoid underprediction which is not suited for R^2^ optimisation. 

### 3.8. Model Evaluation Using the Cosmetics Europe Database

Recently, a dataset was compiled by the Cosmetics Europe consortium to enable comparisons of skin sensitisation DA models [21,22]. The dataset covers in vitro data (DPRA, KeratinoSens^TM^, h-CLAT), LLNA data, and human potency data for 128 substances. To enable a consistent comparison with other DA models, we decided to use the same dataset to evaluate the newly developed DAs. The data were used within the freshly generated DAs of model A and model B, as well as SaferSkin-BN, for comparison. The outcomes are summarised in Table 8 and Table 9. The individual predictions for all compounds from the Cosmetics Europe dataset by the baseline model, as well as model A and model B, can be found in Supplementary Materials Table S5.

Considering the overall predictions of the complete set (N = 122), model B’s accuracy was superior (68%) to both model A’s performance and that of the baseline model, SaferSkin-BN (63% and 61% accuracy, respectively). The database was originally implemented to evaluate the performance of several DA models by Kleinstreuer et al.: Kao Sequential Testing Strategy, Kao Integrated Testing Strategy, Shiseido Artificial Neural Network (DPRA/h-CLAT or DPRA/h-CLAT/KeratinoSens^TM^), and P&G BN ITS-3 [22]. The resulting accuracies for predicting the LLNA sensitising potency ranged from 65 to 70%. Thus, the accuracies of the newly developed models were of the same order of magnitude as those of the existing DA models. Additional benefits of the newly developed DAs are improved accessibility, transparency, and a more flexible spectrum of in vitro input parameters. Beyond that, our newly developed models A and B provide four-class potency prediction, while the DAs evaluated by Kleinstreuer et al. [22] provide up to three potency classes. This difference needs to be considered when comparing the derived accuracy values. A higher number of potency classes is beneficial for safety assessment since it decreases the range of possible potencies for compounds assigned to a certain potency class.

To predict the three human potency classes, the four LLNA classes were combined into three classes (strong, weak = moderate + weak, non-sensitising), and accuracies were evaluated on the complete set (N = 122), as well as for different confidence levels (Table 9). The predictive capacity for LLNA was superior to the prediction of human potency. This can be explained by the fact that all three models were trained on the LLNA data. LLNA data, despite their variability, are experimental values obtained in reproducible experiments, whereas human potency data are expert gradings that also incorporate the exposure of the population [25]. For the human endpoint, we again observed that the accuracy of model A was comparable to that of the baseline model. In contrast, model B offered a slight improvement over the baseline model. To compare the newly developed models with other DAs, we again turned to the work by Kleinstreuer et al. [22], where DAs were evaluated on the Cosmetics Europe dataset. To briefly summarise the results from [22] on the accuracy of predicting three human potency classes, all DAs performed comparable or better than the LLNA assay (59% accuracy). The worst DA in [22] was the Bayesian network approach with 55% accuracy, the same as the newly developed model A. The best performing DAs were Kao STS and Kao ITS with 64 and 69% accuracy. With model B, we reach the same performance as the neural network approaches by Shiseido ANN (D_hC) and ANN (D_hC_KS) with 61 and 63% accuracy.

An advantage of the presented BN models is their capacity to provide a confidence estimation for a single prediction. For each sensitiser potency class, the prediction probability is calculated based on the evidence from in vitro assays and in silico tools. Consistent results from different in silico and in vitro tools increase confidence, whereas conflicting results decrease confidence. A good correlation between prediction accuracy and the estimated confidence level was observed (Table 8 and Table 9). Predictions with very strong confidence reached accuracies of up to 88%, whereas the predictions with weak confidence were associated with accuracies below 50%. Therefore, predictions with weak confidence should be scrutinised thoroughly and may require an additional safety assessment factor.

### 3.9. Applicability and Limitations of Proposed Approach

Among the strengths of the proposed approach are (i) its ability to handle incomplete input parameters; (ii) its provision of the confidence level of predictions, which are strongly correlated to the model’s accuracy; (iii) and its provision of the EC3 value that is require for risk assessment.

However, the approach also has limitations. One is the discretization of continuous input variables, which will necessarily result in potentially mispredicted compounds that have continuous input parameters close to their discretization thresholds. Such cases should be inspected carefully, and predictions could be generated with slight variation of such input parameters.

Another limitation is that the approach requires a known chemical structure to convert in vitro parameters into molar units. So, the issue of how to handle cases of natural substances with unknown chemical structures is not very well defined.

## 4. Conclusions

In this article, we describe the development of two new DA models, model A and model B, with the aim to increase the applicability and flexibility of current approaches by allowing input from a new in vitro method as well as from freely available in silico tools. Both models developed are based on previously published Bayesian network approaches, ITS-3 and SaferSkin-BN [9,12]. The existing DAs were upgraded by expanding the set of in vitro input parameters with integrated kDPRA data and by increasing accessibility and transparency by replacing the commercial in silico software TIMES-SS and ACD/Labs with the publicly accessible software Skin Doctor CP and OPERA. The most accurate model, model B, includes predictions from the commercial in silico tool Derek Nexus to comply with recommendations for in silico toxicology protocols. The combination of rule-based and statistical in silico prediction tools increased the performance of model B compared with model A and the baseline model SaferSkin-BN, respectively. Using the Cosmetics Europe dataset to evaluate the newly developed models enables direct comparison with other published DA models evaluated on the same dataset [22]. Models A and B both showed comparable or even superior accuracy compared to the baseline model, SaferSkin-BN. A comparative evaluation of published DAs showed similar accuracy at 63% to 68%. The models’ accuracy increased by considering a higher confidence level of up to 85%. However, the newly developed models enable a more granular potency assessment because they predict four instead of three classes. The incorporation of additional LLNA data would further improve the predictive capacity, as well as the confidence in predictions. While the models are trained and optimised for LLNA prediction, we could also show their capability to predict skin sensitisation in humans. In addition, we could show that the incorporated measure of reliability can indicate the model’s applicability to an individual molecule of interest. This would allow us to distinguish between highly and less reliable predictions. Our analysis shows a general trend in potency overestimation for the predicted pEC3 derived from higher percentiles. This overestimation results in a more conservative and, consequently, more protective calculation of safe concentrations using NGRA approaches. Ultimately, this work supports the goal of reaching regulatory acceptance of newly developed DA models for NGRA approaches to skin sensitisation.

## Figures and Tables

**Table 1:** Optimal parameters and balanced accuracies of BN models A and B predicting four potency classes on the test set.

Model Architecture	Optimal Hyperparameters						Balanced Accuracy on Test Set (%)
	N_CYTOX_	N_CYSTEINE_	N_HCLAT_	N_BIOAV_	N_INSILICO_	N_REP_	
Baseline (SaferSkin-BN)	3	4	4	3	N/A	20	66
Model A	3	3	4	3	N/A	20	66
Model B	3	3	3	3	4	20	70

**Table 2:** Balanced accuracies of BN models A and B predicting four potency classes on the test set with and without kinetic DPRA (kDPRA) data.

Model	Balanced Accuracy (%)
Baseline (SaferSkin-BN)	66
Model A	66
Model A without kDPRA nodes	64
Model B	70
Model B without kDPRA nodes	68

**Table 3:** Balanced accuracies of BN models A and B predicting four potency classes with different in silico inputs.

Model	Balanced Accuracy (%)
Baseline (SaferSkin-BN)	66
Variation of model A with TIMES-SS replacing the Skin Doctor CP node	66
Model A (with Skin Doctor CP node)	66
Model B (with Skin Doctor CP and Derek Nexus nodes)	70

**Table 4:** Balanced accuracies of the BN models A and B predicting four potency classes on the test set using physicochemical properties from ACD/Labs and OPERA.

Model	Balanced Accuracy (%)
Baseline (SaferSkin-BN)	66
Model A trained with ACD/Labs phys-chem.	66
Model A trained with OPERA phys-chem.	66
Model B trained with ACD/Labs phys-chem.	69
Model B trained with OPERA phys-chem.	70

**Table 5:** Cross-validated and fold-balanced accuracies of BN models A and B in predicting four potency classes.

Model Architecture	Cross-Validated	Fold-Balanced Accuracy (%)		
		Minimum	Median	Maximum
Model A	66	55	65	83
Model B	68	52	65	90

**Table 6:** Cross-validated balanced accuracies of BN models A and B in predicting four potency classes with different sets of hyperparameters.

Model Architecture	Cross-Validated Balanced Accuracy (%)		
	Minimum	Median	Maximum
Model A	59	64	68
Model B	61	68	75

**Table 7:** Accuracy of BN models A and B on the test set when predicting four potency classes, calculated separately for each confidence class. The number of compounds for each class, N, is provided in parentheses.

Model Architecture	Accuracy (%)			
	Weak Confidence	Substantial Confidence	Strong Confidence	Very Strong Confidence
Model A	48 (N = 33)	55 (N = 69)	55 (N = 31)	88 (N = 69)
Model B	40 (N = 25)	56 (N = 67)	67 (N = 33)	90 (N = 69)

**Table 8:** Accuracy of the baseline and BN models A and B in predicting four LLNA potency classes on the Cosmetics Europe dataset calculated separately for each confidence class. The number of compounds for each class, N, is provided in parentheses.

Model Architecture	Accuracy (%)				
	Complete Dataset	Weak Confidence	Substantial Confidence	Strong Confidence	Very Strong Confidence
Baseline (SaferSkin-BN)	61 (N = 122)	46 (N = 28)	62 (N = 39)	44 (N = 25)	87 (N = 30)
Model A	63 (N = 122)	33 (N = 30)	65 (N = 54)	83 (N = 12)	85 (N = 26)
Model B	68 (N = 122)	43 (N = 37)	72 (N = 43)	86 (N = 22)	85 (N = 20)

**Table 9:** Accuracy of the baseline and BN models A and B in predicting three human potency classes on the Cosmetics Europe dataset calculated separately for each confidence class. The number of compounds for each class, N, is provided in parentheses.

Model Architecture	Accuracy (%)				
	Complete Dataset	Weak Confidence	Substantial Confidence	Strong Confidence	Very Strong Confidence
Baseline (SaferSkin-BN)	57 (N = 122)	54 (N = 28)	44 (N = 29)	64 (N = 25)	70 (N = 30)
Model A	55 (N = 122)	43 (N = 30)	52 (N = 54)	58 (N = 12)	73 (N = 26)
Model B	61 (N = 122)	49 (N = 37)	63 (N = 43)	64 (N = 22)	75 (N = 20)

## Data Availability

All relevant data are within the manuscript and its Supplementary Materials.

## Supplementary Materials

The following supporting information can be downloaded at: https://www.mdpi.com/article/10.3390/toxics12090666/s1, Table S1: Data set used for training of the BN models; Table S2: Data set used for evaluation of the BN models; Table S3: Cosmetics Europe data set enriched with results from kDPRA, Derek, Skin Doctor CP and OPERA as used for further evaluation of the BN models; Table S4a: Predictions on the test set returned by the baseline model; Table S4b: Predictions on the test set returned by model A; Table S4c: Predictions on the test set returned by model B; Table S5a: Predictions on the Cosemtics Europe data set returned by the baseline model; Table S5b: Predictions on the Cosemtics Europe data set returned by model A; Table S5c: Predictions on the Cosemtics Europe data set returned by model B.
